# Transgender and Gender Nonconforming in Emergency Departments: A Qualitative Report of Patient Experiences

**DOI:** 10.1089/trgh.2016.0026

**Published:** 2017-02-01

**Authors:** Makini Chisolm-Straker, Logan Jardine, Cyril Bennouna, Nina Morency-Brassard, Lauren Coy, Maria Olivia Egemba, Peter L. Shearer

**Affiliations:** ^1^ Department of Emergency Medicine, Icahn School of Medicine at Mount Sinai, New York City, New York.; ^2^ Department of Emergency Medicine, SUNY Downstate College of Medicine, Brooklyn, New York.; ^3^ Department of Population and Family Health, Columbia University School of Public Health, New York City, New York.; ^4^ Emergency Response Team, International Rescue Committee, New York City, New York.; ^5^ Obstetrics, Gynecology and Reproductive Services, Bixby Center for Global Reproductive Health, University of California, San Francisco, San Francisco, California.; ^6^ New York City Teens Connection, Center for Health Equity, New York City Department of Health and Mental Hygiene, Queens, New York.

**Keywords:** discrimination, emergency department, emergency medicine, gender nonconforming, patient perception, power, provider training, stigma, transgender

## Abstract

**Background:** Individuals who have a transgender or gender nonconforming (TGGNC) experience belong to a marginalized segment of the U.S. population, and healthcare can be difficult for them to navigate. Although emergency departments (EDs) traditionally serve as healthcare “safety nets” for vulnerable populations, quantitative studies outside the United States have found that TGGNC-experienced persons tend to avoid EDs and/or have negative experiences. This qualitative study primarily describes the ED experiences of people with a TGGNC history; furthermore, the study explores reasons why this population avoids U.S. EDs and their recommendations for improvements to ED care.

**Methods:** This qualitative study used data about TGGNC-historied persons' experiences in U.S. EDs from retrospective, anonymous, written surveys (paper or web based). National data collection took place from June 2012 through December 2014. Participant responses (*n*=240) were examined using thematic analysis.

**Results:** Using a framework that recognized positive and negative responses, the themes of Self-Efficacy and Power Inequity surfaced. These themes exposed the tension between patients with TGGNC experiences and clinicians who were perceived to lack training in this area, resulting in negative patient experiences. When practitioners had specific training about this population, participants reported positive care experiences.

**Conclusions:** This study indicates that many TGGNC-historied persons who use U.S. EDs have negative experiences, largely due to lack of provider sensitivity toward and training about this patient population. Data from this investigation suggest that training of U.S. ED providers and institutional support would help improve care for this marginalized group.

## Introduction

Gender is frequently perceived to be binary and correlative to anatomical sex, but this is not the case for all.^1,2^ Gender is an internal experience and relates to masculinity, femininity, and their various combinations; it can be demonstrated through body language, clothing, voice, and other social and cultural expressions.^3^ Those with a transgender or gender nonconforming (TGGNC) life experience or history^*^ have a gender identity different than the gender assumed at birth.

Individuals with TGGNC life experiences are among the most marginalized and disenfranchised people in the United States: They disproportionately experience homelessness, underemployment, and live in extreme poverty. Furthermore, in one national study, 41% had attempted suicide compared to the general population's rate of less than 2%.^4,5^ Unemployed people with a TGGNC life experience are more likely to perform work in the “underground economy” (e.g., sex work or selling drugs), be incarcerated, or use alcohol or illicit substances to cope with mistreatment experienced; this group (unemployed with a TGGNC history) is four times more likely to be HIV infected.^4^ And in a 2011 national study by the National Center for Transgender Equality, many with a TGGNC experience had survived physical (61%) and sexual (64%) assault.^4^

Multiple studies report that TGGNC-historied people frequently avoid accessing necessary healthcare because of harassment and discrimination in healthcare settings.^4,6–14^ Emergency Departments (EDs) are the de facto “safety net” of the U.S. healthcare system, open to anyone in medical need, at any time.^15^ The Emergency Medicine Treatment and Active Labor Act of 1986 was enacted to prevent clinicians from turning away those who lack insurance or the ability to pay but have emergency medical conditions.^16^ This has resulted in EDs being a setting where anyone can request evaluation and treatment. Thus EDs, which serve many marginalized populations, should also be able to care for patients with a TGGNC history, who are clearly among the nation's most vulnerable.

While 92% of general ED patients in the United States report being satisfied with the emergency care received,^17^ it is unknown how TGGNC-historied persons in the United States experience the nation's EDs, which should be accessible to this vulnerable population, as it is for others. This investigation explores how TGGNC-historied persons, as a disenfranchised group, experience care in the medical safety net of the United States.

## Materials and Methods

“TG in the ED” was an anonymous, retrospective, written survey accessible in English and Spanish. The survey was available online via SurveyMonkey and in paper form. Data collection started in June 2012 and ended in December 2014, when new surveys no longer revealed new themes.

Eligible participants self-identified as having a TGGNC life experience, were at least 18 years old, could read and write in English or Spanish, and wanted, needed, and/or had used an ED in the United States. A convenience sample of participants was recruited from across the United States via community and health centers that serve lesbian, gay, bisexual, and TGGNC-experienced (LGBT) persons; Facebook^†^; a 2014 national conference on transgender health^18^; and word-of-mouth.

The institutional review boards of both The Icahn School of Medicine at Mount Sinai and Columbia University in New York City approved the study. Written and verbal consent was waived to protect the anonymity of participants and no remuneration was provided for participation.

The survey questions were drafted based on the existing literature and refined by TGGNC health experts, including persons with a TGGNC life experience and those providing healthcare to this population for at least 10 years. Free response questions were posed to participants about experiences in the ED, as well as current gender identity, reasons for nonuse of the ED, and recommendations for ED clinicians to improve their care of this patient population.

**FIG. 1.**
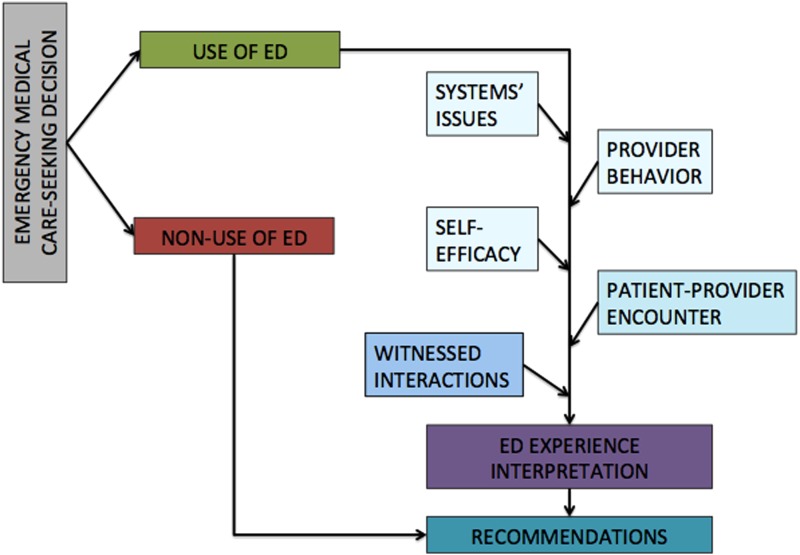
Conceptual framework: Social cognitive theory and stigma theory.

As data were being collected, the research team developed a simple conceptual framework (Fig. 1) based on social cognitive theory (SCT) and stigma theory. These public health theories helped guide analysis of how participants experienced ED care. SCT posits that behavior is influenced by environmental and personal factors, as well as the behavior itself. This model is strongly based on the role of self-efficacy (an individual's belief in their own capacity to execute behaviors necessary to achieve specific goals) in behavior change.^19^ Yet, SCT alone does not account for the patient–provider power differential and its effects on patient experiences. Stigma theory rests on the idea that all social relationships occur within an unequal structure, in which some groups have more social, political, and/or economic power than others; and that traditional power holders may, intentionally or unintentionally, use stigma and discrimination to reinforce existing structural inequities to assert their possession of power.^20^

The primary survey question for those who sought emergency medical care was open ended: “Please tell us about your experience.” To examine the data, investigators collaboratively developed labels, or codes, to apply to lines of text such that similar pieces of information could be grouped and compared. The codes were synthesized into one codebook, with rules on when to use or not use each code. Two expert consultants in qualitative analysis, acquainted with the data, reviewed the codebook and then with the team coded five pages of data to determine utility of codes and finalize the codebook. Codes were applied to the data using NVivo 10.^‡^,^21^

To classify respondents' reports about their experiences in the ED, the principal investigator (MCS) first examined the frequency with which each code was applied, then all text that fell under multiple codes. Next, each ED experience was reviewed for all codes used to describe the experience. For the data display (Table 1), the codes were then grouped according to SCT-influenced categories: the category of “Provider Behavior” included the codes “competency,” “outed,” “provider discomfort,” and “confidentiality.” Under “Patient–Provider Encounter” were the codes “respect,” “gendered,” “inappropriate,” and “perceived medical care.” “Systems Issues” included “institutional practice,” and the category of “Self-Efficacy” incorporated “fear,” “disclosure,” and “self-efficacy.” An experience was considered “negative” if the participant expressed discontent with medical care and/or interactions with ED personnel. When participants described satisfaction with care and/or interactions, a “positive” interaction was inferred.

**Table 1.** **Data Display, Abridged**

Conceptual frame	Code	Positive interpretation	Negative interpretation
Provider behavior	Competency	Participants who reported positive experiences with provider competency noted TGGNC-related training of staff. “I have had positive experiences (in terms of my trans status) in the ER, probably because it is [name of hospital] and they are educated for the most part about trans issues.” R134	Participants stated that their providers did not know how TGGNC life experience is relevant to a chief complaint or how hormone use affects health. “Treated by a resident, who told me ‘I don't understand your body. I will just consider you female, but who knows what those hormones you are taking could be doing to you’.” R155
Patient–provider encounter	Gendered	Some described being properly pronouned or named and feeling respected because of this. “I was actually treated really nicely by the doctors and hospital staff…they treated me like the woman that I am and I would recommend that hospital to anyone who asked.” R86	Participants who described being misgendered mostly noted that even after their gender identity or experience was disclosed they were mispronouned. “It was humiliating because I was repeatedly referred to by my birth sex after repeatedly asking them to call me by male pronouns.” R59
Patient–provider encounter	Inappropriate		This code captured participant descriptions of provider gawking, persistent staff focus on genital examinations, unnecessary history taking about gender-related surgeries, sexual history, assumption of sexually transmitted infections, drug use, psychological disorder, and use of patient for “teaching” that was interpreted as being put on display. “I was…repeatedly asked if I was drunk or on drugs. I was neither. Injury to leg required stitches.” R170
Self efficacy	Self efficacy	Those who had others advocate on their behalf reported experiencing respect and proper pronouning. “Emergency appendectomy needed….both my primary physician and spouse took very good care to ensure I was treated respectfully.” R129	Those who reported being able to advocate for themselves usually did not have a positive experience; others found themselves unable to advocate for themselves at all. “I had a baby and probably could have used some gender related support from a licensed professional during the delivery, but was too frightened to ask for it.” R18

A summary of the context in which each code was found was grouped into the “positive” and/or “negative” column of the data display with illustrative participant quotes. Some codes were only negative, so no positive data were found there.

TGGNC, transgender or gender nonconforming.

As a team of six investigators with diverse qualities, it was important to recognize how the investigators' identities and experiences might shape the analysis and bias findings. Collectively, the team accounted for these subjectivities by working independently first and then coming together, with the consultants, to review strengths and limitations of the separate efforts. For example, the consultants appreciated that most codes were initially biased toward reflecting negative experiences. Hence, many such codes were changed, before being applied to the data, to be neutral, such that positive experiences could be recognized.

“Member checking”^22^ was performed, to determine whether the investigators' interpretations of these data reflected the experiences of TGGNC-historied persons receiving emergency care in the United States. Advertised for 6 months on Facebook, this anonymous validation survey was completed by five English-literate, self-identified TGGNC-experienced individuals, some of whom may have participated in the original survey. These persons were given a brief summary of the findings and asked to anonymously report, via SurveyMonkey, whether they considered the results accurate.

## Results

Two hundred forty participants completed the main survey; Table 2 describes the demographics and medical characteristics of the sample. Table 3 describes reasons respondents reported for not using EDs; the most common explanations were previous negative experiences or fear of experiencing discrimination.

**Table 2.** **Participant Characteristics**

	%	*n*=240
Age (in years)		
18–24	17.5	42
25–35	50.4	121
36–45	16.7	40
46–55	9.2	22
56–64	3.8	9
≥65	0.8	2
Annual income (in USD)		
<11,000	16.3	39
11,001–22,000	15.4	37
22,001–35,000	15.4	37
35,001–45,000	10.8	26
45,001–55,000	7.9	19
55,001–70,000	12.1	29
70,001–90,000	6.7	16
90,001–100,000	4.2	10
>100,000	9.2	22
Highest level of education completed		
Junior high/middle school	1.3	3
High school	6.3	15
Some college/university	22.1	53
College/university	35.4	85
Certificate program	2.1	5
Graduate/professional school	31.3	75
Race and/or ethnicity^a^		
Asian/Asian American	4.2	10
African American/black	3.8	9
Latino/Hispanic	10.4	25
Native American/Amerindian/Alaskan Native/Pacific Islander	2.1	5
White	82.5	198
Other	2.5	6
Sex assigned at birth		
Female	60.4	145
Male	21.7	52
Other	0.8	2
Ever-used gender-affirming hormones		
No	22.1	53
Yes	65.4	157
Not applicable^b^	2.1	5
Prescribed by a licensed provider (*n*=157)		
No	3.8	6
Yes	95.5	150
At least one gender-affirming surgery		
No	38.3	92
Yes	51.7	124
Have a primary care provider		
No	17.1	41
Yes	72.5	174
Ever wanted/needed to use ED		
No	13.3	32
Yes	75	180
Went to ED (*n*=180)		
No	8.3	15
Yes	90.6	163

^a^ Respondents were able to choose more than one category.

^b^ Respondents with a gender nonconforming experience may not desire hormone use.

ED, emergency department.

**Table 3.** **Reasons for Transgender or Gender Nonconforming Nonuse of U.S. Emergency Departments**

Conceptual frame	Reason	Frequency (*n*=35),^a^ % (*n*)
Personal	Lack of medical insurance	31.4 (11)
	Fear of being outed, misgendered, or experiencing discrimination	60 (21)
Provider behavior	Past witnessing of medical personnel gossiping, mocking, or telling jokes about the TGGNC patients	45.7 (16)
Patient–provider encounter	Past experience of being purposely outed by healthcare professional	8.6 (3)
	Past experience with visibly uncomfortable providers and/or being asked inappropriate questions	34.3 (12)
	Past experience of staff refusal to use preferred pronouns	62.9 (22)
	Past experience of transphobia as a patient	37.1 (13)
Systems issues	Medical facilities are unable to provide accommodations for TGGNC patients	42.9 (15)
	Providers are poorly educated in TGGNC health-related issues	40 (14)

^a^ Participants who answered this question could offer multiple reasons. Recommendations were included here if they resonated with responses to other questions, not based on frequency to one question.

**FIG. 2.**
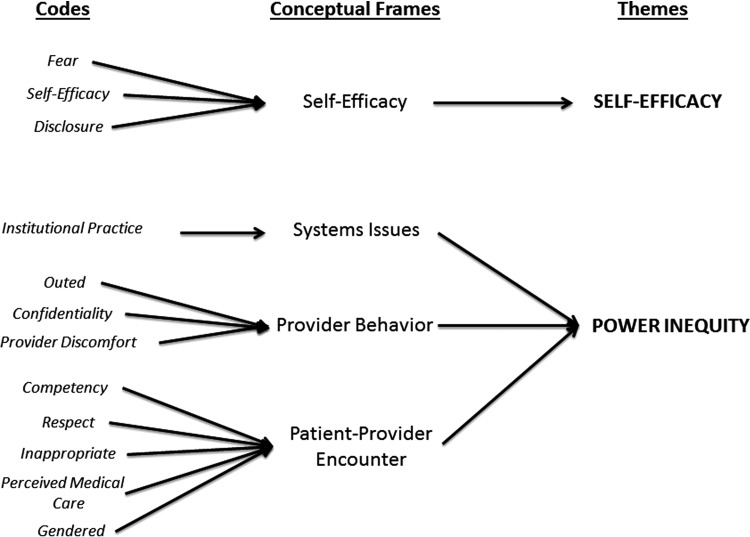
Thematic analysis.

Two overarching themes emerged from the codes related to experiences of emergency care (Fig. 2). The conceptual frame of “Self-Efficacy” allowed the larger theme of *Self-Efficacy* to materialize; and the conceptual frames “Provider Behavior,” “Patient–Provider Encounter,” and “Systems Issues” were encompassed by the stigma theory-influenced theme of *Power Inequity*. These two themes guided the analysis toward an appreciation of the altered power dynamic between providers and patients that results from TGGNC-historied patients presenting to EDs. All validation surveys confirmed the interpretation that regardless of intent, the providers' efforts to compensate for inexperience with TGGNC-related health issues and to assert themselves as knowledgeable authorities led to negative experiences for many study participants.

### Self-efficacy

The code “disclosure” centered on participants' discussions about considerations of divulging their TGGNC experience to ED staff. Whether forced or elective, “disclosure” was generally linked to a perception of poor medical care. One respondent recounted,

…I revealed my status, which no one knows usually until I tell them. And then things got weird. The doctors were very rude, barely treated me, and tried to get rid of me as quickly as possible. And worst of all, when I tried to use the woman's restroom before I left, they threatened to call security on me. It was humiliating. I would die before I went back there again (R21).

Some participants disclosed their TGGNC history and reported being treated with respect, which most attributed to the staff being well trained: “I have had positive experiences (in terms of my trans status) in the ER probably because…they are educated for the most part about trans issues” (R133). Another participant's positive experience was attributed to having a support network advocating for quality care: “…both [my] primary physician and spouse took very good care to ensure I was treated respectfully” (R127). Some reported not disclosing and feeling respected because they could pass within the gender binary or they were unsure if their clinician(s) were aware of their TGGNC history.

The “self-efficacy” code was used when participants spoke up for themselves, with resultant positive or negative experiences, and also when participants purposefully had someone else advocate on their behalf. Some found that the traditional provider–patient hierarchy barred their self-efficacy. About a portion of the physical examination that may have been unnecessary, one participant said, “I didn't ask at the time because…I have been taught that doctors know better than I do” (R118). However, even those who reported being able to advocate for themselves often did not have a positive experience:

I always remind them when I check in that I am trans (FTM^§^). I would rather providers feel uncomfortable before they see me and then get their s—- together by the time they come in. Even though I do that, the providers always ask me questions about my penis and fail to ask important questions pertinent to people with female anatomy until I prompt them to.… (R123)

Those who had others advocate on their behalf experienced respect and proper pronouning. “My experience was generally positive, but I made sure to have a friend come with me. The doctors referred to me by [desired] female pronouns…” (R148).

### Power inequity

Many of the negative experiences described by participants included difficulties with practitioner knowledge in treating TGGNC-experienced patients. Generally, providers did not seem to know when and how TGGNC-related medical history was relevant to a chief complaint: “Doctor unable to consider that my female history and male hormones could impact my health in the situation presented” (R132). Participants conveyed expecting providers to be unaware and reported having to explain the medical relevance of TGGNC medical histories. When positive experiences were noted, they were attributed to purposeful training: “Once I spoke with the Dr. the whole team was great. I later learned my primary care Dr. had worked at that hosp. prior, and had explained gender issues with much of the senior staff” (R25).

Respondents noticed that in situations in which their clinicians admitted they were not knowledgeable about TGGNC-related health issues, these providers were rude and unprofessional. One participant reported that the nurses “…[said] things like how it was against God and just wasn't right” (R82). Providers who demonstrated discomfort with this patient population commonly misgendered patients: “Referred to as a woman even after I explained to the doctor that I was a transmale, they ignored my statement and proceeded to call me she” (R210).

In addition to misgendering patients, having insufficient medical knowledge, and visibly expressing discomfort, clinicians were often perceived to perform unnecessary histories and physical examinations. One participant reported being “questioned a lot about my sex life when I went for pneumonia” (R62). Participants described experiencing open gawking, superfluous history taking about gender-related surgeries, assumption of sexually transmitted infections, drug use, and psychiatric disease, and being put on display. One participant shared, “I have also had doctors/nurses call over other people on duty to come look at me for no reason. It made me feel like an animal in a zoo” (R208). Participants also reported having their gender “variance” verbally or physically exposed in the waiting area in front of other patients: “One visit required an ekg, and after telling the staff I was trans, they continued to open my shirt with the doors and curtains open to the ER waiting room” (R10).

Moreover, failure of practitioners to communicate led participants to assume they had received an inappropriate examination. For example, one participant survived a motor vehicle collision and experienced head and neck trauma. The participant reported the provider

…[felt] parts of my lower body (through my clothes) that seemed to have nothing to do with my head/neck/spine. He specifically felt where my hips were, using both hands to register where they were. To this day I don't know if this was medically necessary or if he was medically curious about my transsexuality. (R118).

Most likely, this participant is describing the basics of a secondary survey, in which the clinician assesses pelvis stability, but in the absence of communication, the patient inferred the worst.

To improve ED care, participants recommended that hospitals develop TGGNC-sensitive protocols and practitioner trainings to ensure privacy, respect, and appropriate treatment for patients (Table 4). They recommended focusing on the application of gendered language and improving both intake procedures and modes of communication that currently risk unnecessarily exposing a patient's TGGNC experience.

**Table 4.** **Participant Recommendations to Improve the Emergency Department Care of Persons with a Transgender or Gender Nonconforming Experience**

Conceptual frame	Recommendation	Frequency (123),^a^ % (*n*)
Provider behavior	Do not discuss gender identity or TGGNC experience (e.g., gender-affirming surgeries) with others, including healthcare providers, unless it is relevant to provision of care.	22.8 (28)
	Ask sensitive questions in private spaces only.	5.7 (7)
	Call out last names only (no prefixes) in group areas, such as waiting rooms.	2.4 (3)
Patient–provider encounter	Standard practice of providers should be to ask patients' preferred pronoun and name and use these throughout care.	44.7 (55)
	Do not ask about gender and/or TGGNC experience if it is not relevant to ED care.	35.8 (44)
	Avoid gender-specific terms, including social titles (eg: Ms., Mr.).	1.6 (2)
Systems issues	Systematic, required training of ED providers on TGGNC medical issues, including gender-affirming surgeries, potential postoperative complications, common hormone therapies, and related side effects. Providers should also be trained on the social stigma and marginalization this population experiences generally and in healthcare settings.	13.8 (17)
	Incorporate pronoun and name preference in registration forms.^b^	9.8 (12)
	Train clinicians on how to ask, when clinically relevant, about sensitive gender information.	4.1 (5)
	Offer gender-neutral spaces, including hospital rooms and restrooms.^c^	2.4 (3)

^a^ Participants could offer more than one recommendation. Recommendations were included here if they resonated with responses to other questions, not based on frequency to one question.

^b^ While most who commented on pronoun preference on registration forms were in favor of this addition, some were concerned about the potential ramifications of a TGGNC experience being in their medical record.

^c^ Applicable to all areas of the hospital, not just emergency departments.

## Discussion

While many factors impact patient satisfaction with ED experiences, clinician knowledge is not usually identified as a key area for improvement.^17,23–27^ However, participants in this study reported having to tell their practitioners if and how their TGGNC medical history was relevant to the presenting complaint. This lack of practitioner knowledge on the issue was strongly tied to negative experiences for TGGNC-experienced patients. Further research should examine the curricula of professional healthcare schools and EDs that provide TGGNC health training to students and staff and explore efforts to systematize such education.

Particularly concerning among the negative experiences, multiple participants reported experiencing unwanted examinations, similar to the qualitative findings from a European study.^9^ Even when a clinician considers an examination necessary, patients with capacity have the right to refuse examinations or treatment plans.^28–30^ Examinations performed out of provider desire to learn more about TGGNC-related medicine are appropriate only with patient permission. When practitioners believe an examination (or history element) is relevant, it is particularly important to explain the clinical reasoning to patients of marginalized populations, including TGGNC-experienced patients. Not communicating why an examination (or history element) is relevant to care alienates this vulnerable and high-risk group. Similar to TGGNC-historied reports of general healthcare avoidance,^2,4,7,9–14^ many participants in this ED-focused study indicated that they have avoided ED care due to fear of or previous experience of unwanted examinations.

The negative experiences reported additionally included inappropriate history taking, illness assumption, and misgendering. TGGNC-historied persons have also described these experiences in general medicine reports in Europe, New Zealand, and in some U.S. states.^2,4,7,9–14^ Such staff actions lead to TGGNC-experienced patients, even those who have not used the ED but have heard of such interactions, avoiding needed medical care (Table 3).^2,4,7,9–14^ More research is needed to explore why patients who had a cisgender^**^ advocate had more positive ED experiences than those who advocated for themselves; similarly, more research should examine why TGGNC-historied patients who appeared cisgender had a better ED experience than patients who did not.

### Limitations

As a survey promoted by organizations and clinics that serve LGBT populations, this study has limitations. Respondents had relatively high educational and income levels; they may differ from those without access to primary care or kin advocates. Insurance status was not systematically assessed in this study, although several participants noted that not having insurance or the gender marker with their insurance impacted their decision to seek ED care. Further research should explore whether insurance status and insurance gender markers have any impact on ED care experiences among TGGNC-historied persons. Despite specific attempts to optimize diversity, respondents are largely white and were designated “female” at birth. As with any voluntary survey, persons with negative experiences may be more likely to participate than those with positive experiences, and here they may be more likely to attribute their negative experience to a TGGNC history. Nevertheless, the negative experiences reported are concerning and largely elucidate the findings of previous quantitative studies.^4,12^ The written, anonymous format permitted a large, national sample but barred discussion with participants. Still, this study, the first of its kind in the United States, situates the responses within a framework of social theory and contributes to a richer understanding of what shapes the ED experiences of TGGNC-historied persons.

## Conclusion

As the frontline of protection for the nation's public health, U.S. ED providers must be prepared to serve the most vulnerable and high-risk populations: ED visits can be unique opportunities to link marginalized patients to needed social and medical resources with the purpose of comprehensively improving the health of communities.^15^ This study demonstrates that TGGNC-historied patients in the United States have negative and alienating ED experiences, which they attribute to lack of provider training on care provision for this population. Table 5 lists resources clinicians may find useful in learning more about TGGNC healthcare. Further research is needed to examine interventions that may strengthen EDs' ability to function as the safety net for this socially and economically marginalized group with significant health concerns.

**Table 5.** **Educational Resources**

“Affirmative Care for Transgender and Gender Non-Conforming People: Best Practices for Front-line Healthcare Staff,” by the National LGBT Health Education Center. Available at: www.lgbthealtheducation.org/wp-content/uploads/13-017_TransBestPracticesforFrontlineStaff_v9_04-30-13.pdf
“Understanding Transgender: Frequently Asked Questions about Transgender People,” by the National Center for Transgender Equality. Available at: http://transequality.org/issues/resources/understanding-transgender-people-faq
“Standards of Care for the Health of Transsexual, Transgender, and Gender-Nonconforming People.” *World Professional Association for Transgender Health*, 7th Version, 2012. Available at: www.wpath.org
“Transgender Patients in the ED.” *Emergency Physicians Monthly, August 19, 2015.* Available at: http://epmonthly.com/article/transgender-patients-in-the-ed/

## Contributor Acknowledgments

*Qualitative research consultants:* Marina Catallozzi, MD, Timothy D. Cunningham, MSN, RN; *Survey Development:* Gavriel Ansara, PhD, MSc, Maddie Deutsch, MD, Alex Gonzalez, MD, MPH, Eva Hersch, MD, Ruben Hopwood, MDiv, PhD, Asa Radix, MD, MPH, Lynne D. Richardson, MD, Norman Spack, MD; *Recruiting Organizations:* Callen-Lorde Community Health Center, New York; Gay Central Valley, California; GLBT Advocacy and Youth Services, Inc., Alabama; Mazzoni Family and Community Medicine, Pennsylvania; Oasis Youth Center, Washington; SAGE Metro, Michigan; The Gay & Lesbian Community Center, Pennsylvania.

## Abbreviations Used

EDs: Emergency Departments
SCT: social cognitive theory
TGGNC: transgender or gender nonconforming

## Notes

^*^ Transgender “life experience” and “history,” instead of transgender “identity,” is used in this article based upon validation survey feedback from experienced leaders in the TGGNC health field: “Transgender” describes an experience, not necessarily an identity. It was noted that “being transgender” leaves out people who simply identify as one gender, but were formerly recognized as another. For example, one might identify as male, but was once recognized as female; such a man may not identify as “transgender,” only as male. “History” and “life experience” were suggested for use as more encompassing of the ways in which people understand themselves.

^†^ www.Facebook.com/TGinED

^‡^ NVivo is software designed to help qualitative investigators organize, conceptualize, and understand raw, unstructured data such as participant survey responses.

^§^ FTM: Female to Male.

^**^ “Cisgender” describes a person whose performed gender conforms to his or her assigned gender at birth.
